# One‐Pot Catalytic Conversion of Lignin‐Derivable Guaiacols and Syringols to Cyclohexylamines

**DOI:** 10.1002/cssc.202200914

**Published:** 2022-08-01

**Authors:** Xianyuan Wu, Mario De bruyn, Katalin Barta

**Affiliations:** ^1^ Stratingh Institute for Chemistry University of Groningen Groningen The Netherlands; ^2^ Department of Chemistry, Organic and Bioorganic Chemistry University of Graz Heinrichstrasse 28/II 8010 Graz Austria

**Keywords:** amines, catalytic amination, cyclohexylamines, reductive catalytic fractionation, sustainable chemistry

## Abstract

Cyclic primary amines are elementary building blocks to many fine chemicals, pharmaceuticals, and polymers. Here, a powerful one‐pot Raney Ni‐based catalytic strategy was developed to transform guaiacol into cyclohexylamine using NH_3_ (7 bar) and H_2_ (10 bar) in up to 94 % yield. The methodology was extendable to the conversion of a wider range of guaiacols and syringols into their corresponding cyclohexylamines. Notably, a crude bio‐oil originating from the reductive catalytic fractionation of birch lignocellulose was transformed into a product mixture rich in 4‐propylcyclohexylamine, constituting an interesting case of catalytic funneling. The isolated yield of the desired 4‐propylcyclohexylamine reached as high as 7 wt % (on lignin basis). Preliminary mechanistic studies pointed at the consecutive occurrence of three key catalytic transformations, namely, demethoxylation, hydrogenation, and amination.

## Graphical abstract

**All in one**: A one‐pot catalytic methodology enables the production of industrially relevant cyclohexylamines in good to excellent yields over Raney Ni catalyst/*t‐*amyl alcohol, using NH_3_ and H_2_ at 180 °C, and generating water and methanol as the only side products.

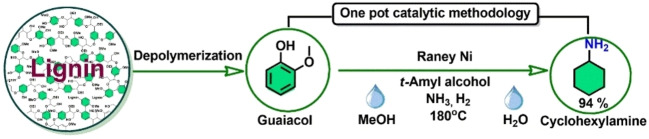

## Introduction

Cyclohexylamine and its derivatives are industrially important building blocks for the manufacturing of pharmaceuticals, agrochemicals, textiles, electronics, and vulcanization accelerators.[^1^, ^2^, ^3^, ^4^, ^5^] Cyclohexylamine is mainly produced from non‐renewable petroleum‐derived nitrobenzene and aniline, and to a lesser extent through the amination of phenol with ammonia.[^6^, ^7^] The overall strong reliance of cyclohexylamine production on benzene, irrespective the route, poses potential future challenges as benzene is derived from finite fossil resources, be it crude oil or natural gas, or mainly as a by‐product from steam crackers, oil refineries, and *p*‐xylene production, which strongly links its availability to the overall gasoline, ethylene, and *p*‐xylene demand. Additionally, styrene and ethylbenzene production take up more than half of the total benzene production.^[8]^ Further strain derives from a tight benzene supply/demand and rising benzene prices.^[9]^

For all the above outlined reasons, a strong interest exists for the development of green and sustainable catalytic pathways to the formation of cyclohexylamine and its derivatives.[^10^, ^11^, ^12^, ^13^, ^14^, ^15^, ^16^] Presently, many sustainable catalytic routes to the formation of generic primary amines have been established,[^17^, ^18^, ^19^, ^20^, ^21^, ^22^, ^23^, ^24^, ^25^, ^26^, ^27^, ^28^, ^29^] some of which effectively start from lignocellulose‐derived platform molecules.[^23^, ^24^, ^25^, ^26^, ^27^, ^28^, ^29^] Exemplary are (a) the Ru−MgO/TiO_2_‐catalyzed amination of furfuryl alcohol to furfuryl amine using ammonia,^[30]^ (b) the Ni/Al_2_O_3_‐ and Rh/C‐catalyzed near‐quantitative formation of cyclohexylamine from phenol and ammonia,[^13^, ^14^] and (c) the Ni/SiO_2_−Al_2_O_3_‐catalyzed amination of vanillyl alcohol into its corresponding amine in 58 % yield by our group, via a hydrogen‐borrowing approach.^[31]^ However, very limited research is available on the transformation of lignin‐derived guaiacyl and syringyl substrates to cyclohexylamines, which would overall constitute a markedly more sustainable process.[^32^, ^33^, ^34^] Such transformation would involve two consecutive steps: the simultaneous demethoxylation/hydrogenation of guaiacols to cyclohexanols, and the direct amination of said cyclohexanols with ammonia to their corresponding cyclohexylamines.[^11^, ^35^, ^36^, ^37^] Particularly challenging is the development of an efficient and highly selective one‐pot process.[^13^, ^34^]

Reductive catalytic fractionation (RCF) is a well‐developed, highly efficient methodology for the depolymerization of lignin.[^38^, ^39^, ^40^, ^41^] More specifically, it has been shown to yield mixtures of 4‐propylguaiacol/syringol and 4‐propanolguaiacol/syringol in near‐theoretical yields.[^42^, ^43^, ^44^] However, there is a lack of an efficient catalytic downstream processing strategy that enables funneling of such phenolic mixtures into higher‐value products due to the complexity of the crude lignin oil.[^45^, ^46^]

**Figure 1:**
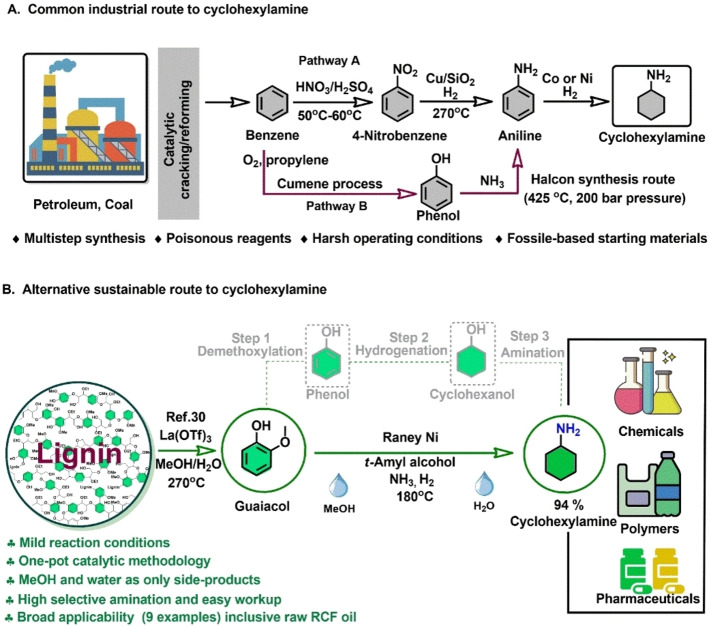
An overview towards the production of cyclohexylamine. (A) Current industrial pathway to produce cyclohexylamine. (B) One‐pot, three‐step, reductive catalytic demethoxylation/hydrogenation/amination of lignin‐derived guaiacol (exemplary) into cyclohexylamine in *t*‐amyl alcohol over a Raney Ni catalyst.

Herein, we present a three‐step Raney Ni‐based catalytic methodology for the conversion of lignin‐derived guaiacols and syringols into cyclohexylamines in one‐pot. Excellent yields (up to 94 %) of the latter compounds are paired with a low environmental footprint, the side products being only methanol generated from demethoxylation and water obtained from the amination step (Figure 1B). Moreover, in starting from birch wood lignin, it is shown that the consecutive application of Ru/C (RCF step) and Raney Ni (this reductive amination methodology) allows to obtain 4‐propylcyclohexylamine in up to 6.7 wt % isolated yield on a lignin basis.

## Results and Discussion

### Transformation of guaiacol to cyclohexylamine

**Figure 2:**
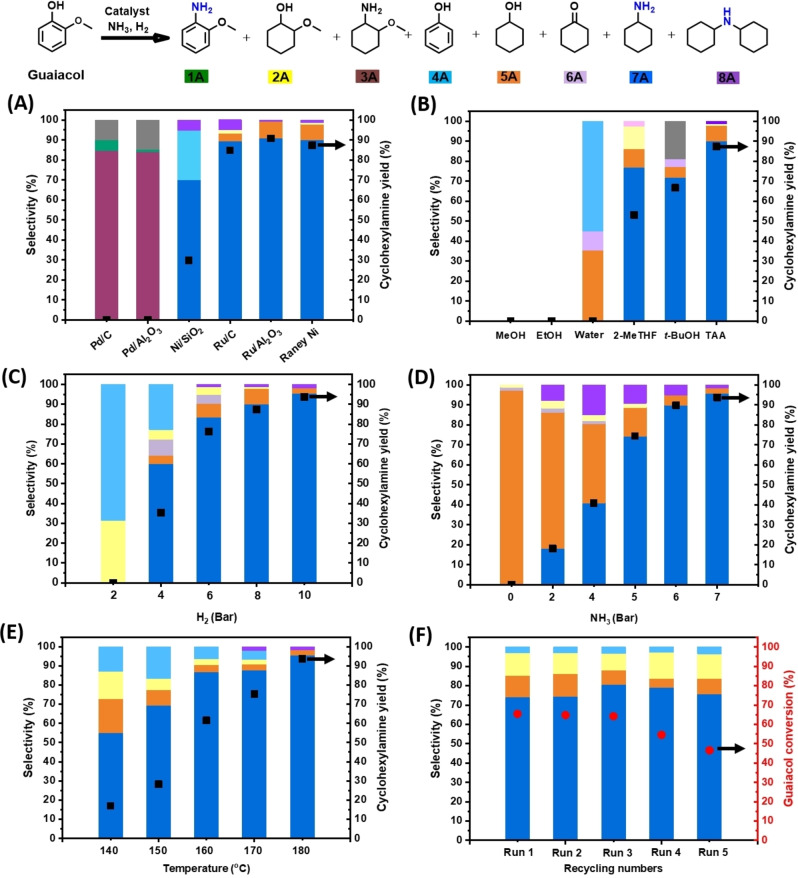
Influence of a range of reaction parameters on the conversion of guaiacol to **7A**. The effective reaction conditions are outlined in the accompanying Supporting Information Tables to these graphs; in very general terms: 0.5 mmol guaiacol, 200 mg catalyst, 3 mL solvent, 180 °C, 0–7 bar NH_3_, 2–10 bar H_2_, 20 h for (A–E) and 4 h for (F), 5 mg dodecane as the internal standard. Conversion, selectivity, and yield values determined by gas chromatography flame ionization detector (GC‐FID) using calibration curves and internal standard. In the graphs, the black square symbol indicates the **7A** yield, while the red circle symbol stands for the conversion of guaiacol.

In a first phase, a range of commercially available heterogeneous catalysts were evaluated in the one‐pot catalytic conversion of guaiacol to cyclohexylamine as a model reaction under a standard set of reaction conditions. It can be inferred from Figure 2A (Table S1) that Pd/C and Pd/Al_2_O_3_ were excellent catalysts for the hydrogenation and amination of guaiacol, yielding 2‐methoxycyclohexanamine (**3A**) in over 84 % yield. In line with the existing literature, the latter heterogeneous Pd catalysts did not engage in demethoxylation and thus did not further convert **3A** into cyclohexylamine (**7A**).^[34]^ Changing the active metal to ruthenium, as with Ru/C and Ru/Al_2_O_3_, afforded the formation of **7A** from guaiacol in 84.8 and 90.8 % yield, respectively. Usage of Ni/SiO_2_ as the catalyst led to the formation of **7A** in only 29.7 % yield, the main side product being phenol (**4A**). In using Raney Ni, however, this could be significantly improved to an overall **7A** yield of 87.3 %. This suggests a unique capability of Raney Ni to perform not only hydrogenation but also demethoxylation, as was also shown by recent work of Wang and Rinaldi, and alcohol amination, which is in line with previous work by our group.[^31^, ^36^, ^47^, ^48^, ^49^] We attribute the high required catalyst loading to the presence of inactive Ni oxide in Raney Ni as well as inaccessible Ni in the bulk of the catalyst. Also, commercial Raney Ni is typically characterized by the presence of a substantial amount of water (≈20 wt %).

Having identified Raney Ni as the best‐performing non‐noble catalyst, the influence of a range of reaction solvents was investigated (Figure 2B, Table S2). While *t*‐amyl alcohol (TAA) was found to be an excellent solvent for this reaction (Figure 2A, Table S1), the alternative use of methanol or ethanol reduced the activity of the Raney Ni catalyst to near zero. (Figure 2B, Table S2). The latter observation was in line with findings by De Vos and co‐workers on Ni/Al_2_O_3_‐mediated reductive amination of phenol,^[13]^ where the detrimental effect of alcoholic solvents on the catalyst activity was attributed to a strong interaction between the OH groups and the catalyst and/or a higher ammonia solubility, which could equally negatively affect the catalysis.[^13^, ^36^, ^50^] However, in marked contrast, the here presented reductive amination reactions run smoothly in the presence of TAA. This may be attributed to less favorable adsorption of TAA, as a tertiary alcohol, onto the active sites of Raney Ni compared to the runs in which Ni/Al_2_O_3_ is used. Interestingly, in the presence of water, the reaction was found limited to demethoxylation (**4A**) and (consecutive) hydrogenation to afford cyclohexanol (**5A**) and cyclohexanone (**6A**), with no amination products being observed. Changing TAA for *tert*‐butylalcohol (*t‐*BuOH), thus keeping the tertiary alcohol moiety in the solvent, led to a marked decrease in the **7A** yield, going from 87.3 to 66.7 %, respectively. Employing 2‐methyltetrahydrofuran, a cyclic ether in this reaction, led to a further decrease of the **7A** yield to 53.1 %.

For a given NH_3_ pressure (7 bar) and reaction time (20 h), also the influence of the hydrogen partial pressure was investigated (Figure 2C, Table S3). At 2 bar H_2_ partial pressure, 13.1 % of the original guaiacol was converted, yielding **4A** and 2‐methoxycyclohexanol (**2A**) in 68.6 and 31.4 % selectivity, respectively. Using 4 bar H_2_ partial pressure shifted the selectivity away from **2A**/**4A** to **4A** (22.7 %) and to **7A** (60 %). Still higher H_2_ partial pressures (6–10 bar) gave increasingly higher **7A** yields (up to 93.7 %). The observed different catalytic behavior at low hydrogen partial pressure (2 bar) is likely related to lower hydrogenation rate under these conditions and may also be additionally caused by a stronger adsorption of NH_3_ onto the Ni catalytic active sites, leading to an inhibiting effect during the demethoxylation and hydrogenation of guaiacol.[^13^, ^51^]

For a given reaction time and H_2_ pressure (10 bar) we also investigated the influence of the ammonia partial pressure (Figure 2D, Table S4). In the absence of ammonia, **5A** was found to be the major and expected reaction product (97.1 % yield). Applying increasing NH_3_ partial pressures (2–6 bar) increased the formation of the target product **7A** at the expense of **5A** formation. At 7 bar NH_3_ partial pressure, **7A** was formed in 93.7 % yield. Overalkylation to dicylohexylamine (**8A**) was only detected at lower NH_3_ partial pressures, which is in line with previous results on catalytic amination by our group.[^31^, ^49^]

At the optimal NH_3_ and H_2_ partial pressures (7 and 10 bar, respectively), and for a given reaction time (20 h), the influence of temperature on catalytic activity and selectivity was investigated. It can be seen from Figure 2E (Table S5) that the conversion of guaiacol and the **7A** yield improved with increasing temperature, the highest **7A** yield being 93.7 % at 180 °C. Recycling of the Raney Ni catalyst was achieved by magnetic separation from the reaction mixture, a proven methodology.[^31^, ^52^] As can be inferred from Figure 2F (Table S6), the obtained **7A** yield was found largely constant over the first two recycles. From the third cycle onward, a moderate decrease of the conversion level was observed (at constant **7A** selectivity), thus decreasing the overall **7A** yield. Inductively coupled plasma (ICP) analysis of the reaction media after removal of the Raney Ni catalyst revealed no detectable Ni leaching (Table S7). With optimized reaction conditions, finally, the influence of catalyst loading on reductive amination of guaiacol has also been investigated (Table S8). It was found that the optimal catalyst loading was 200 mg, leading to highest 93.7 % yield to **7A**. While Using 50 or 100 mg catalyst loading gave poorer reactivity, with only 47.2 and 64.2 % yield to **7A** obtained, respectively. Thus, high loading of catalyst is necessary to achieve the desired yield to **7A** from guaiacol.

**Figure 3:**
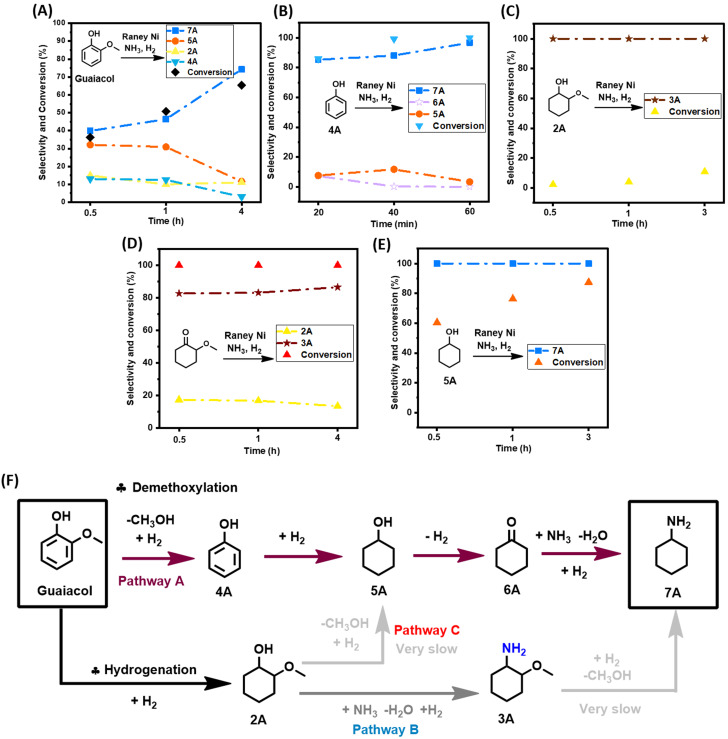
Tentative mechanistic proposal. (A) Summary of the main overall reaction. (B–E) Reactivity studies on selective model compounds. (F) Proposed reactive network to the direct conversion of guaiacol into **7A**. Reaction conditions: 0.5 mmol guaiacol, 200 mg catalyst, 3 mL *t‐*amyl alcohol, 5 mg dodecane as the internal standard, 180 °C,7 bar NH_3_, 8 bar H_2_. (C,D) Conversion and selectivity were determined by GC‐FID.

From a mechanistic point of view, the first step in the transformation of guaiacol to **7A** could be guaiacol demethoxylation (pathway A) and/or guaiacol hydrogenation. In combination with the above discussed experimental data (as summarized in Figure 3A), the observation that phenol (=demethoxylated guaiacol) can be rapidly converted into 7 A in 96.7 % in 1 h reaction time (Figure 3B) lends credibility to pathway A.

However, subjecting **2A** (=hydrogenated guaiacol) to the here developed catalytic methodology (Figure 3C), and respecting the same 1 h reaction time frame, was found to effectively convert **2A** to **3A** (amination reaction) at full selectivity yet at low **2A** conversion. The comparatively slow transformation of **2A** to **3A** (vis‐à‐vis pathway A), combined with the absence of **7A** in the reaction mixture, strongly disfavors pathway B. The observed high selectivity for **3A** also precludes the occurrence of a hybrid pathway where guaiacol hydrogenation is followed by **2A** demethoxylation to **5A** (pathway C), then entering pathway A. It is equally noteworthy that the data displayed in Figure 2 show no **3A** accumulation, further disfavoring pathway B. Rapid demethoxylation of the aromatic guaiacyl/syringyl motifs, as well as the much lower rate of demethoxylation once the aromatic guaiacyl/syringyl units are hydrogenated, has also been observed by our group when investigating the reactivity behavior of other aromatic substrates.[^48^, ^49^]

Interestingly, under different reaction conditions, De Vos and co‐workers have investigated the reductive amination of guaiacol with Ni/Al_2_O_3_ or Rh/C yielding 2‐methoxycyclohexylamine in 4 and 57.6 %, respectively.[^13^, ^14^] Thereby the poor results obtained with the Ni/Al_2_O_3_ catalyst were attributed to complexation of Ni by guaiacol, resulting in the formation of a blue–green reaction mixture, and hence Ni‐leaching.^[13]^ In this work, owing to the nature of the Raney Ni catalyst, neither green–blue discoloration of the reaction medium nor Ni leaching was observed. Furthermore, the specific absence of pathway B contrasts to a certain degree with the literature observation that demethoxylation of 3‐methoxycyclohexanone is possible via β‐elimination,[^13^, ^17^] that way forming cyclohexylamine via 2‐cyclohex‐1‐one, albeit under slightly different conditions. The latter intermediate could potentially also be reached by the α‐demethoxylation of 2‐methoxycyclohexanone (partial hydrogenation of guaiacol). Applying though the here developed catalytic amination methodology on 2‐methoxycyclohexanone does neither yield 2‐cyclohex‐1‐one nor cyclohexylamine (Figure 3D).

In a further advancement, the validity of the developed catalytic procedure was also tested on a range of lignin‐derived guaiacols and syringols (Table 1). Within the guaiacol series, ethyl guaiacol also gave a high GC and isolated yield (87.1 and 80.6 %, respectively) of its corresponding saturated amine, 4‐ethylcyclohexylamine. Most other alkylated guaiacols gave GC and isolated yields at the 70 and 50 % level, respectively. Applying the newly developed catalytic methodology to syringol gave cyclohexylamine in 71 % GC yield. This is important as natural lignin contains both guaiacyl and syringyl units, the relative ratios of the latter two units being dependent on the plant species.^[43]^

**Table 1:** Applicability of the developed amination methodology to a range of lignin‐derived guaiacols/syringols.^[a]^

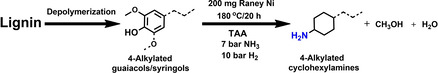					
Entry	Substrate	Product	Conv. [%]	GC yield (isolated yield) [%]	*cis*/*trans* ratio [%]
1	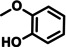	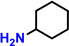	98.0	93.7 (82.1)	–
2	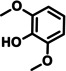		94.6	71.0	–
3	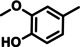	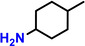	93.4	76.2 (55.6)	32 : 68^[b]^
4	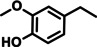	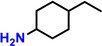	99.0	87.1 (80.6)	12 : 88
5	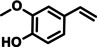		78	56.6 (47.7)	–
6	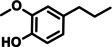	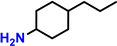	96.8	71.6 (50.9)	11 : 89
7	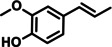		97.0	71.2 (46.0)	–
8	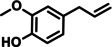		94.5	72.4 (46.4)	–
9	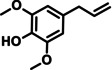		55.2	34.6	–

[a] Reaction conditions: 0.5 mmol substrates, 200 mg catalyst, 3 mL *t‐*amyl alcohol, 180 °C, 7 bar NH_3_, 10 bar H_2_, 20 h. [b] *cis*/*trans* isomers were determined by commercial compound.

**Figure 4:**
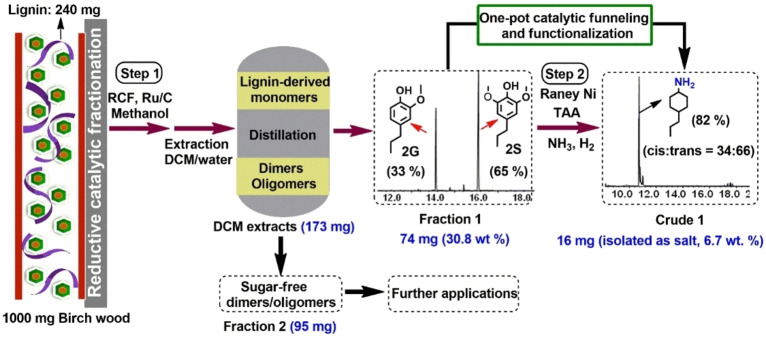
Two‐step catalytic procedure to the production of 4‐propylcyclohexylamine from native birch lignocellulose. Step 1 (RCF procedure): 1000 mg birch wood, 150 mg Ru/C catalyst, 20 mL methanol, 220 °C, 30 bar H_2_, 18 h. Step 2 (amination procedure): 74 mg distilled monomers, 500 mg Raney Ni, 3 mL TAA, 7 bar NH_3_, 10 bar H_2_, 48 h. The GC yield values are shown in black while the isolated yield values are depicted in blue.

In this respect we have also applied the developed catalytic methodology to a crude bio‐oil obtained from RCF of birch lignocellulose (Figure 4).^[42]^ More specifically, RCF of 1 g birchwood in the presence of a Ru/C catalyst at 30 bar H_2_ in methanol and 220 °C gave crude depolymerized lignin oil, which was treated by a suitable extraction and washing procedure to yield organic extracts. The extracts were vacuum‐distilled (1 mbar) at 220 °C for 0.5 h, yielding Fraction 1 (74 mg, 30.8 wt % yield to monomers) that constitutes a mixture of the two well‐defined aromatic monomers, 2‐methoxy‐4‐propyl‐phenol (**2G**) (33 %) and 2,6‐dimethoxy‐4‐propyl‐phenol (**2S**) (65 %) (Figure S7). Application of the here developed amination methodology to Fraction 1 gave a crude product rich in 4‐propylcyclohexylamine (82 % selectivity) and with a *cis*/*trans* ratio of 1 : 2 (Figure S8). Isolation of the latter amine as pure compound was achieved as its HCl salt (6.7 wt % on a lignin basis).

To evaluate the greenness of the here developed process, we have included a CHEM 21 comparative metrics analysis at the first path level of the here presented Raney Ni‐catalyzed guaiacol to cyclohexylamine transformation versus a modified version of the industrial Halcon phenol to aniline/cyclohexylamine process (Supporting Note 1, Scheme S1).[^53^, ^54^, ^55^] The results of this CHEM21 analysis are summarized in Tables S9 and S10. From the latter Table, it can be inferred that the here developed methodology is significantly superior to the modified Halcon process since the second step of the modified Halcon process is particularly underperforming with clear red flags on selectivity and yield. These red flags are mainly attributed to the distinct formation of dicyclohexylamine and aniline as side products. On the other side, the atom economy (AE) of our developed Raney Ni‐catalyzed method is lower than seen with the modified Halcon process, a matter which relates to the necessary loss of the methoxy group in guaiacol. It can, however, be argued that the side formation of methanol is beneficial as it has a distinct industrial value. Likewise, the loss in reaction mass efficiency (RME) (75.1 %) relates mainly to the loss of guaiacol's methoxy group. Nonetheless, the RME of the Raney Ni process is still superior to the one of the modified Halcon process and then especially when considering the poor RME of the second step in the modified Halcon process. The process mass intensity (PMI) of our Raney Ni process is significantly higher than the ones calculated for the modified Halcon process, a matter which relates entirely to the high amount of TAA used in the Raney Ni process. It is, however, noteworthy that the hazard codes assigned to TAA do not yield red flags in the CHEM21 analysis. Also, TAA is easily derivable from biomass in multiple reaction steps by catalytic methods (Supporting Note 2).

Furthermore, the higher amount of catalyst used negatively influences the PMI of the reagents. It is further noteworthy that the modified Halcon process is operated in the gas phase without the involvement of solvents. Both processes use catalysts (green flag), but the modified Halcon processes has the advantage that it is performed in flow (green flag), whereas the Raney Ni process features batch operation (yellow flag). From a health and safety perspective, though, the modified Halcon process performs much poorer than the Raney Ni one as in addition to the use of H_2_ (highly explosive) and NH_3_ (environmental implications), the phenol substrate and the aniline and dicyclohexylamine by‐products are toxicologically and environmentally unsustainable.

## Conclusion

This work presents a one‐pot catalytic methodology for the production of industrially relevant cyclohexylamines in good to excellent yields. The developed Raney Ni‐based methodology constitutes a feasible avenue to the making of cyclic aliphatic amines directly from renewable resources. Most interestingly, the here presented Raney Ni methodology differs from previous reported excellent amination work using Ni/Al_2_O_3_ and Pd/C in that it also allows for an additional and highly effective demethoxylation step, the relevance of which lies in funneling capability of guaiacyl and syringyl units into one single product, which is important when treating biomass‐derived product streams. Nonetheless, the high catalyst loading still needs to be addressed in future investigations. To the best of our knowledge, the here presented one‐pot catalytic methodology, as well as the high yield of cyclohexylamine from guaiacol, has never been reported. Mechanistically, it was concisely shown that demethoxylation precedes the hydrogenation and amination steps. Finally, it was shown that the developed methodology can transform a crude lignin oil, comprising two monomeric compounds **2G** and **2S**, into 4‐propylcyclohexylamine as a single product and with minimal purification.

## Experimental Section

### Materials and chemicals

2‐Methoxy‐4‐propylphenol (≥99 %), 4‐allyl‐2,6‐dimethoxyphenol (≥95 %), isoeugenol (98 %), eugenol (99 %), 4‐ethylguaiacol (≥98 %), 2‐methoxy‐4‐vinylphenol (≥98 %), 2‐methoxy‐4‐methylphenol (≥98 %), 2,6‐dimethoxyphenol (≥98 %), cyclohexanol (99 %), cyclohexanone (99.8 %), cyclohexylamine (99.9 %), 2‐methoxycyclohexanone (97 %), *t*‐amyl alcohol (99 %), and Raney Ni 2800 were purchased from Sigma‐Aldrich. Guaiacol (>98 %) and phenol (≥99.5 %) were purchased from TCI.

### Catalytic amination of crude lignin oil to 4‐propylcyclohexylamine from birch lignocellulose

**Step 1**: Mild depolymerization of birch lignocellulose was carried out in a high‐pressure Parr autoclave (100 mL) equipped with an overhead stirrer, and this in accordance to a literature procedure.^[39]^ Typically, the autoclave was charged with 150 mg of Ru/C catalyst, 1 g of birch lignocellulose, and 20 mL methanol as the solvent. The reactor was sealed and flushed with H_2_ and pressurized with 30 bar H_2_ at room temperature. The reactor was heated to 220 °C and stirred at 400 rpm for 18 h. After completion of the reaction, the reactor was cooled to room temperature. The solid was separated from the solution by centrifugation and subsequent decantation and additionally washed with methanol (3×20 mL). The methanol washings were combined in a round‐bottom flask, and the solvent was removed under vacuum.

**Extraction procedure**: To the obtained crude mixture, 50 mL DCM and 30 mL water were added, and the resulting mixture was stirred at room temperature for 30 min. The suspension was then transferred into a 100 mL separating funnel to collect the DCM extracts. The latter extracts were dried over anhydrous MgSO_4_. After filtration, the DCM solution was transferred to a round‐bottom flask, and the solvent was removed under vacuum.

**Distillation procedure**: The DCM extract was distilled at 220 °C for 30 min using a Kugelrohr apparatus under a 1 mbar vacuum to give two distinct fractions: the distilled low‐molecular‐weight monomers (Fraction 1) and non‐volatile high‐molecular‐weight dimers and oligomers (Fraction 2).

**Step 2**: The catalytic reductive amination of Fraction1 into 4‐propylcyclohexylamine over Raney Ni catalyst was performed in a high‐pressure Parr autoclave (25 mL) equipped with a magnetic stirrer bar. Typically, a reactor insert tube was charged with 500 mg Raney Ni catalyst, 74 mg Fraction 1, and 3 mL *t‐*amyl alcohol. Then, the vial was placed inside the autoclave after which the reactor was sealed and pressurized with 7 bar NH_3_ and 10 bar H_2_. The reactor was heated to 180 °C and stirred at 400 rpm for 48 h. After completion of the reaction, the reactor was cooled down to room temperature. The overall mixture was resolubilized in 20 mL diethyl ether, followed by the addition of 0.5 mL 2 n HCl in diethyl ether. The resulting ammonium salt was isolated by filtration and washed with diethyl ether (3×20 mL).

### Recycling test of Raney Ni catalyst

The reusability of Raney Ni catalyst was performed under optimized reaction conditions: 200 mg Raney Ni catalyst, 0.5 mmol guaiacol, 3 mL *t*‐amyl alcohol, 5 mg dodecane, 180 °C, 4 h. After the first run, the Raney Ni was magnetically separated from the reaction mixture and washed with 3 mL *t*‐amyl alcohol for 3 times. Then 0.5 mmol guaiacol and 5 mg dodecane were re‐introduced to carry out the next run. After having completed several runs, the solution was combined and concentrated under reduced pressure. The crude mixture was characterized by ICP analysis to determine potential Ni leaching.

## Conflict of interest

The authors declare no conflict of interest.

1

## Supporting information

As a service to our authors and readers, this journal provides supporting information supplied by the authors. Such materials are peer reviewed and may be re‐organized for online delivery, but are not copy‐edited or typeset. Technical support issues arising from supporting information (other than missing files) should be addressed to the authors.

Supporting Information

## Data Availability

The data that support the findings of this study are available in the supplementary material of this article.
