# Supplementary material for: One‐Pot Catalytic Conversion of Lignin‐Derivable Guaiacols and Syringols to Cyclohexylamines
Source: ChemSusChem. 2022 Aug 1;15(18):e202200914. doi: 10.1002/cssc.202200914 (PMC9796232; doi:10.1002/cssc.202200914)
Supplement: Supplementary file 1 — Supporting Information [file CSSC-15-0-s001.pdf]

ChemSusChem

Supporting Information

One-Pot Catalytic Conversion of Lignin-Derivable Guaiacols and Syringols to Cyclohexylamines

Xianyuan Wu, Mario De bruyn, and Katalin Barta*This publication is part of a Special Collection highlighting "The Latest Research from our Board Members". Please visit the Special Collection at .© 2022 The Authors. ChemSusChem published by Wiley-VCH GmbH. This is an open access article under the terms of the Creative Commons Attribution License, which permits use, distribution and reproduction in any medium, provided the original work is properly cited.

Table of Contents

1. General information 3

2. Establishing the optimal reaction conditions for the catalytic conversion of guaiacol to cyclohexylamine 4

3. Assigned GC-FID traces of the catalytic conversion of guaiacol to cyclohexylamine over Raney Ni. 7

4. Supporting ^{1}H spectra 8

5. Assigned GC-FID traces of Fraction 1 and Crude 1 10

Supplementary Note 1: CHEM21 evaluation of the greenness of the here developed process vis-à-vis an industrially relevant process to cyclohexylamine 11

Supplementary Note 2: Synthesis of biomass-derived *t*-amyl alcohol 13

Reference 13

1. General information

Column chromatography was performed using Merck silica gel type 9385 230–400 mesh and typically dichloromethane and methanol or EtOAc and pentane as eluent.

Thin layer chromatography (TLC) Merck silica gel 60, 0.25 mm. The components were visualized by UV or KMnO4 staining.

Gas Chromatography (GC) was used for product identification as well as the determination of conversion and selectivity values. Product identification was performed by a GC-MS (Model: 5975C MSD) equipped with an HP-5 MS column, and helium as the carrier gas. Temperature program: 50 °C for 5 min - 10 $°C \cdot min^{-1}$ to 325 °C - hold for 5 min. Conversion and product selectivities were determined by GC-FID (Agilent 8890 GC) equipped with an HP-5MS column and using nitrogen as the carrier gas.

Nuclear Magnetic Resonance (NMR) spectroscopy: ^1H, and ^{13}C NMR spectra were recorded on a Bruker Avance III 300 MHz (300 and 75 MHz, respectively) and 2D NMR spectra were recorded on a Bruker Avance III 700 MHz equipped from a 5mm Triple-Resonance cryoprobe (700 and 175 MHz, respectively). All NMR spectra were recorded at RT. Chemical shift values are reported in ppm with the solvent resonance as the internal standard ($CDCl_3$: 7.26 for ^1H, 77.0 for ^{13}C; CD_3OD: 3.31 for ^1H, 49.0 for ^{13}C; DMSO-d_6: 2.50 for ^1H, 39.5 for ^{13}C). Data are reported as follows: chemical shifts, multiplicity (s = singlet, d = doublet, t = triplet, q = quartet, br. = broad, m = multiplet), coupling constants (Hz), and integration.

Inductively coupled plasma mass spectrometry (ICP-MS) was performed on an Agilent 7900 ICP-MS. Typically, the samples were solubilized with 5 mL HNO_3 in the MLS ultraclave and then heated to 250 °C for 30 mins before analysis by **ICP-MS.**

Conversion, selectivity and yield calculation

For catalytic demethoxylation/hydrogenation/amination of guaiacol to cyclohexylamine:

$$\text{Conversion (\%)} = \frac{\text{Mol of (original guaiacol} - \text{remaining guaiacol)}}{\text{mol of original guaiacol}} \times 100\ \% \quad \text{Eq 1}$$

$$\text{Selectivity (\%)} = \frac{\text{Mol of the obtained cyclohexylamine}}{\text{Mol of (original guaiacol} - \text{remaining guaiacol)}} \times 100\ \% \quad \text{Eq 2}$$

$$\text{Yield (\%)} = \text{Conversion (\%)} \times \text{Selectivity (\%)} \quad \text{Eq 3}$$

For the catalytic demethoxylation/hydrogenation/amination of 4-propylguaiacol and syringols derived from lignin to 4-propyl cyclohexylamine:

$$\text{Yield (wt\%)} = \frac{\text{Weight of 4} - \text{propylcyclohexylamine}}{\text{Weight of original lignin}} \times 100\ \text{wt\%} \quad \text{Eq 3}$$

2. Establishing the optimal reaction conditions for the catalytic conversion of guaiacol to cyclohexylamine

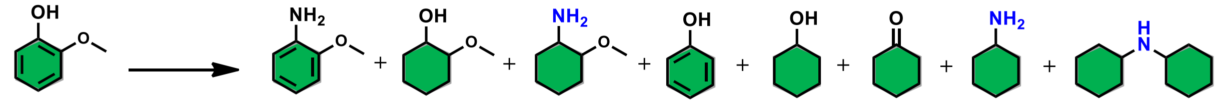

Guaiacol 1A 2A 3A 4A 5A 6A 7A 8A

Table S1. Survey of the activity of a range of commercially available catalysts for the catalytic conversion of guaiacol to cyclohexylamine[a]

Catalyst	Conv.[b] (%)	Selectivity (%)[b]								**7A** Yield[b] (%)
		**1A**[c]	**2A**	**3A**[c]	**4A**	**5A**	**6A**	**7A**	**8A**	
Pd/C	100	5.3	-	84.7	-	-	-	-	-	84.7
Pd/Al_2O_3	100	1.1	-	84.1	-	-	-	-	-	84.1
Ni/SiO_2	42.4	-	-	-	24.7	-	-	70.1	5.2	29.7
Ru/C	94.9	-	1.9	-	-	3.8	-	89.4	5.0	84.8
Ru/Al_2O_3	100	-	-	-	-	8.5	-	90.8	0.7	90.8
Raney Ni	97.0	-	0.8	Trace	-	8.0	0	90.0	1.2	87.3

[a] Reaction conditions: Guaiacol (0.5 mmol), catalyst (200 mg), 180 °C, 20 h, 7 bar NH_3, 8 bar H_2, 3 mL *t*-amyl alcohol, 5 mg dodecane; [b] Conversion, selectivity and yield values were determined by GC-FID using calibration curves and an internal standard; [c] The selectivities to **1A** and **3A** were determined from the GC-FID peak area.

Table S2. Solvent influence in the catalytic conversion of guaiacol to cyclohexylamine over Raney Ni[a]

Solvent	Conv.[b] (%)	Sel. (%) [b]						**7A** Yield (%)
		**2A**	**4A**	**5A**	**6A**	**7A**	**8A**	
Methanol	0	0	0	0	0	0	0	0
Ethanol	0	0	0	0	0	0	0	0
Water	20.0	0	54.9	35.5	9.7	0	0	0
2-MeTHF	69.0	11.2	0	9.3	0	77.0	0	53.1
t-BuOH	92.8	0	0	5.3	3.9	71.9	0	66.7
TAA	97.0	0.8	0	8.0	0	90.0	1.2	87.3

[a]. Reaction conditions: guaiacol (0.5 mmol), 200 mg Raney Ni catalyst, 180 °C, 20 h, 7 bar NH_3, 8 bar H_2, 3 mL solvent, 5 mg dodecane; Conversion, selectivity and yield values are determined by GC-FID using calibration curves and an internal standard.

Table S3. Influence of the hydrogen partial pressure on the catalytic conversion of guaiacol to cyclohexylamine over Raney Ni[a]

Hydrogen (Bar)	Conv.[b] (%)	Sel. (%) [b]						**7A** Yield (%)
		**2A**	**4A**	**5A**	**6A**	**7A**	**8A**	
2	13.1	31.4	68.6	0	0	0	0	0
4	58.8	4.9	22.7	4.2	8.2	60	0	35.3
6	91.2	4.1	0	6.9	4.3	83.6	1.1	76.2
8	97.0	0.8	0	8.0	0	90.0	1.2	87.3
10	98.0	0	0	2.7	0	95.6	1.7	93.7

[a]. Reaction conditions: guaiacol (0.5 mmol), 200 mg Raney Ni catalyst, 180 °C, 20 h, 7 bar NH_3, 2-10 bar H_2, 3 mL *t*-amyl alcohol, 5 mg dodecane; Conversion, selectivity and yield values are determined by GC-FID using calibration curves and an internal standard.

Table S4. Influence of the ammonia partial pressure on the catalytic conversion of guaiacol to cyclohexylamine over Raney Ni[a]

Ammonia (Bar)	Conv.[b] (%)	Sel. (%) [b]						**7A** Yield (%)
		**2A**	**4A**	**5A**	**6A**	**7A**	**8A**	
0	100	1.2	0	97.1	1.7	0	0	0
2	100	3.8	0	68.2	2.1	18.1	7.8	18.1
4	100	3.1	0	39.7	1.4	40.9	14.9	40.9
5	100	2	0	14.4	0	74.3	9.3	74.3
6	100	0	0	5.1	0	89.8	5.1	89.8
7	98.0	0	0	2.7	0	95.6	1.7	93.7

[a]. Reaction conditions: guaiacol (0.5 mmol), 200 mg Raney Ni catalyst, 180 °C, 20 h, 10 bar H_2, 0-7 bar NH_3, 3 mL *t*-amyl alcohol, 5 mg dodecane; Conversion, selectivity and yield values are determined by GC-FID using calibration curves and an internal standard.

Table S5. Influence of the reaction temperature on the catalytic conversion of guaiacol to cyclohexylamine over Raney Ni[a]

Temperature (°C)	Conv. [b] (%)	Sel. (%) [b]						**7A** Yield (%)
		**2A**	**4A**	**5A**	**6A**	**7A**	**8A**	
140	30.8	14.3	12.8	17.8	0	55.1	0	17.0
150	40.8	6.0	16.6	8.1	0	69.3	0	28.3
160	70.8	3.0	6.3	3.7	0	86.9	0	61.5
170	86.0	2.5	4.7	3.2	0	87.7	2.0	75.4
180	98.0	0	0	2.7	0	95.6	1.7	93.7

[a]. Reaction conditions: guaiacol (0.5 mmol), 200 mg Raney Ni catalyst, 140-180 °C, 20 h, 7 bar NH_3, 10 bar H_2, 3 mL *t*-amyl alcohol, 5 mg dodecane; Conversion, selectivity and yield values are determined by GC-FID using calibration curves and internal standard.

Table S6. Recycling tests for the catalytic conversion of guaiacol to cyclohexylamine over Raney Ni[a]

Run	Conv. [b] (%)	Sel. (%) [b]						**7A** Yield (%)
		**2A**	**4A**	**5A**	**6A**	**7A**	**8A**	
Run 1	65.4	11.1	3.0	11.6	0	74.3	0	48.6
Run 2	64.8	11.8	3.0	10.7	0	74.4	0	48.2
Run 3	64.3	7.3	3.3	8.6	0	80.7	0	51.9
Run 4	54.6	4.5	2.8	13.7	0	79.1	0	43.2
Run 5	46.6	8.1	3.7	12.8	0	75.6	0	35.2

[a]. Reaction conditions: guaiacol (0.5 mmol), 200 mg Raney Ni catalyst, 180 °C, 4 h, 7 bar NH_3, 10 bar H_2, 3 mL *t*-amyl alcohol, 5 mg dodecane; Conversion, selectivity and yield values are determined by GC-FID using calibration curves and internal standard.

The Ni leaching contents in the liquid solution (filtrate) was analyzed by Inductively coupled Plasma Mass Spectrometry (ICP-MS). As shown in **Table S7**, there is no obvious Ni leaching after five consecutive runs, thus indicating a good stability of the employed catalytic system.

Table S7. Ni leaching content as analyzed by ICP-MS

Catalytic system	Leached Ni from the catalyst/%	Ni concentration in filtrate/ppm (mg L^{-1})	Ni amount in the filtrate/mg
Raney Ni (200 mg, 20 % water)	0.06	335	0.104

Table S8. Influence of catalyst loading on the catalytic conversion of guaiacol to cyclohexylamine over Raney Ni[a]

Catalyst Loading	Conv.[b] (%)	Sel. (%) [b]						**7A** Yield (%)
		**2A**	**4A**	**5A**	**6A**	**7A**	**8A**	
50	47.2	18.1	0	37.3	0	44.6	0	21.1
100	95.0	3.6	0	28.3	0	67.6	0.5	64.2
200	98.0	0	0	2.7	0	95.6	1.7	93.7

[a]. Reaction conditions: guaiacol (0.5 mmol), 50-200 mg Raney Ni catalyst, 180 °C, 20 h, 10 bar H_2, 7 bar NH_3, 3 mL *t*-amyl alcohol, 5 mg dodecane; Conversion, selectivity and yield values are determined by GC-FID using calibration curves and an internal standard.

3. Assigned GC-FID traces of the catalytic conversion of guaiacol to cyclohexylamine over Raney Ni.

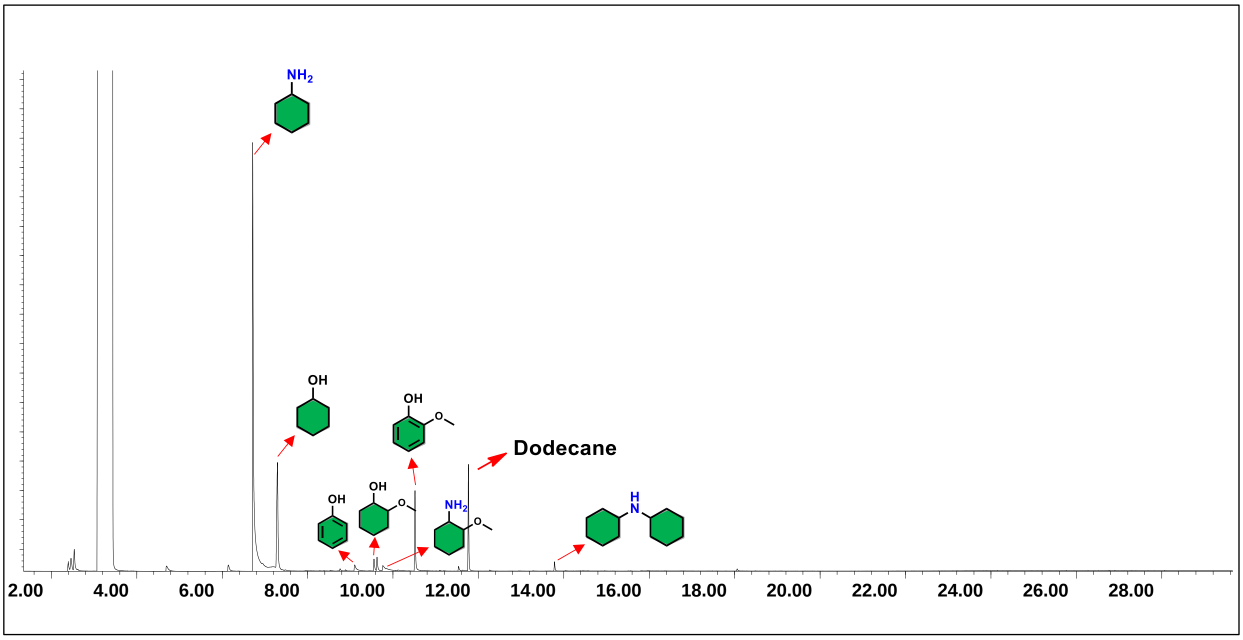

Figure S1. GC-FID spectrum of the catalytic conversion of guaiacol to cyclohexylamine over Raney Ni. Reaction conditions: guaiacol (0.5 mmol), 200 mg Raney Ni catalyst, 170 °C, 7 bar NH_3, 10 bar H_2, 3 mL *t*-amyl alcohol, 5 mg dodecane.

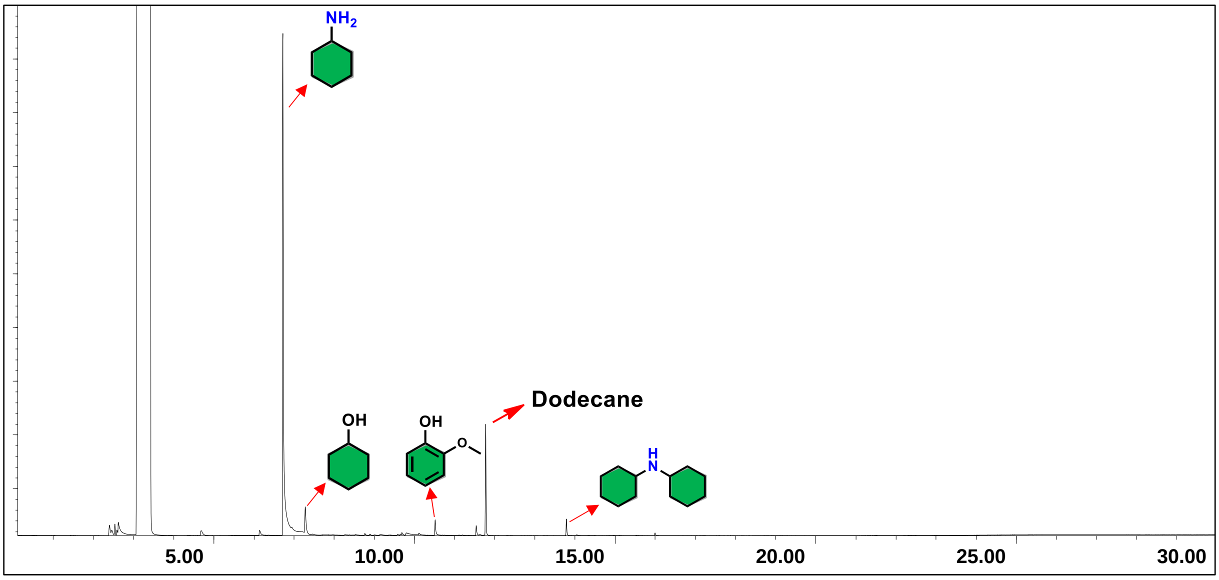

Figure S2. GC-FID spectrum of the catalytic conversion of guaiacol to cyclohexylamine over Raney Ni. Reaction conditions: guaiacol (0.5 mmol), 200 mg Raney Ni catalyst, 180 °C, 7 bar NH_3, 10 bar H_2, 3 mL *t*-amyl alcohol, 5 mg dodecane.

4. Supporting ^{1}H spectra

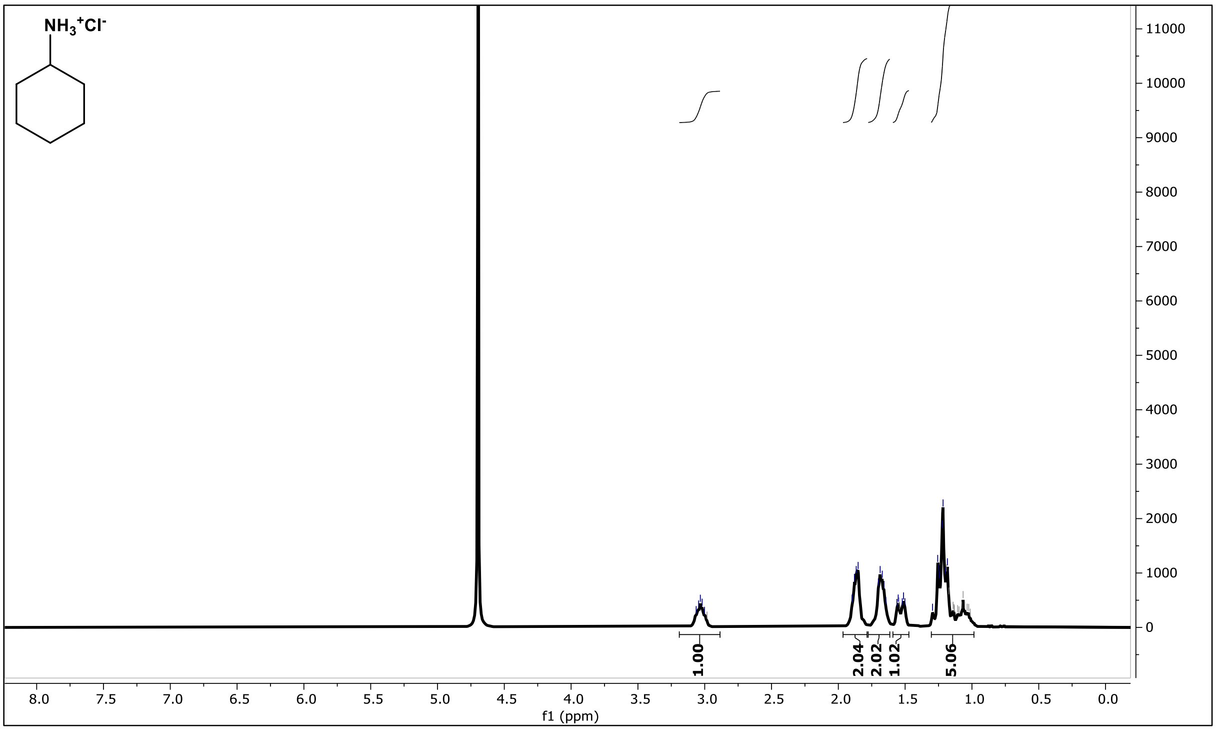

Figure S3. ^{1}H NMR spectrum of cyclohexylamine (as its HCl salt).

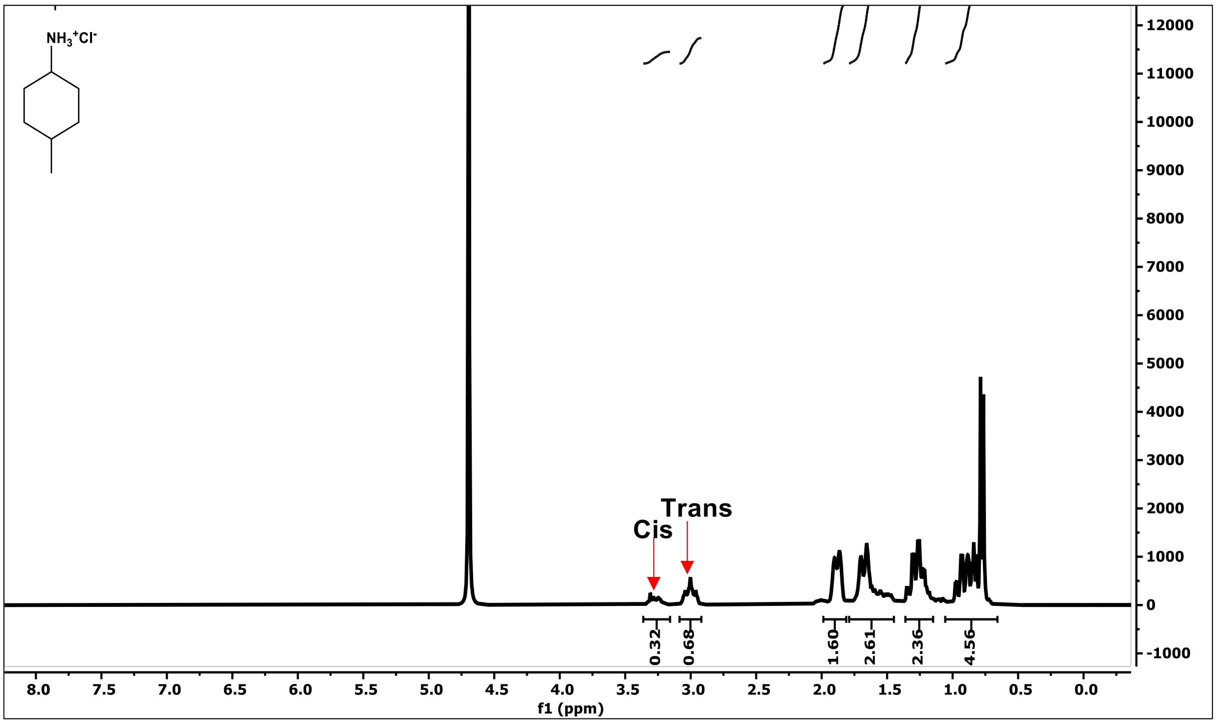

Figure S4. ^{1}H NMR spectrum of 4-methylcyclohexylamine (as its HCl salt).

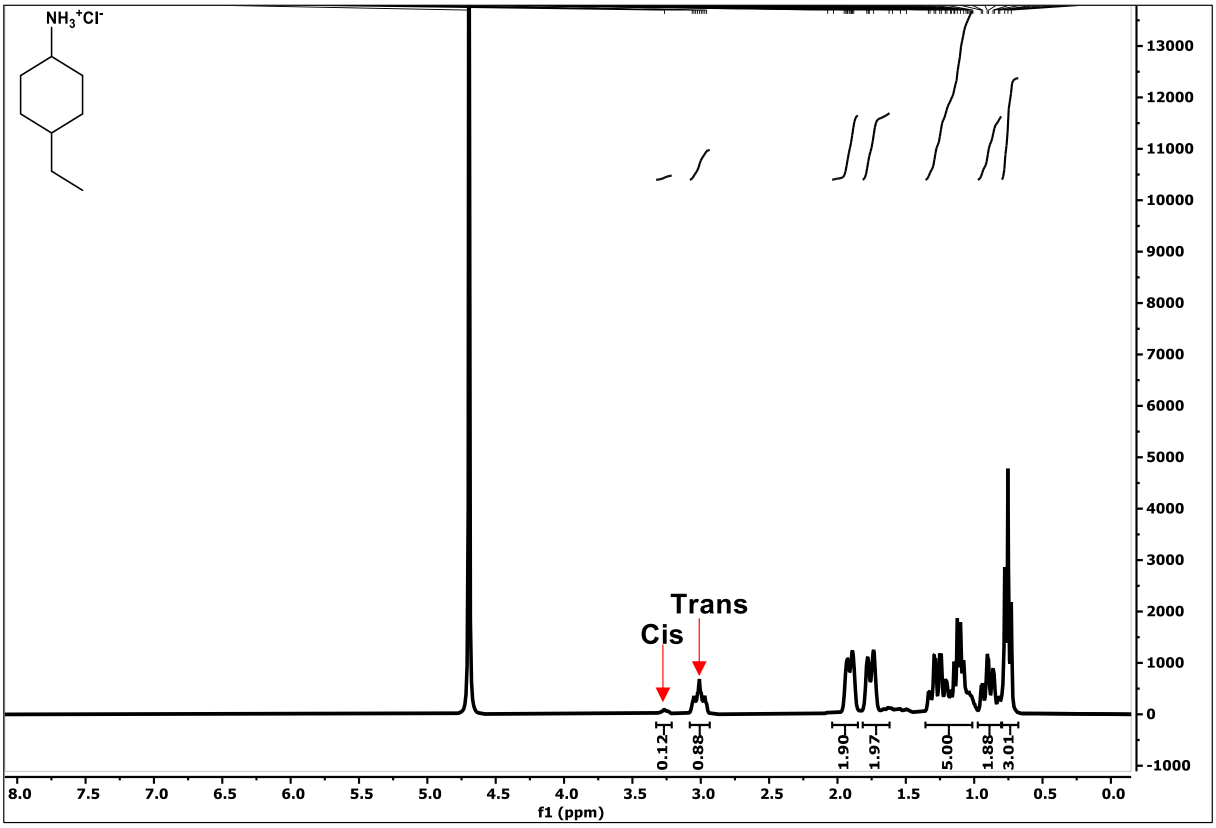

Figure S5. ^{1}H NMR spectrum of 4-ethylcyclohexylamine (as its HCl salt).

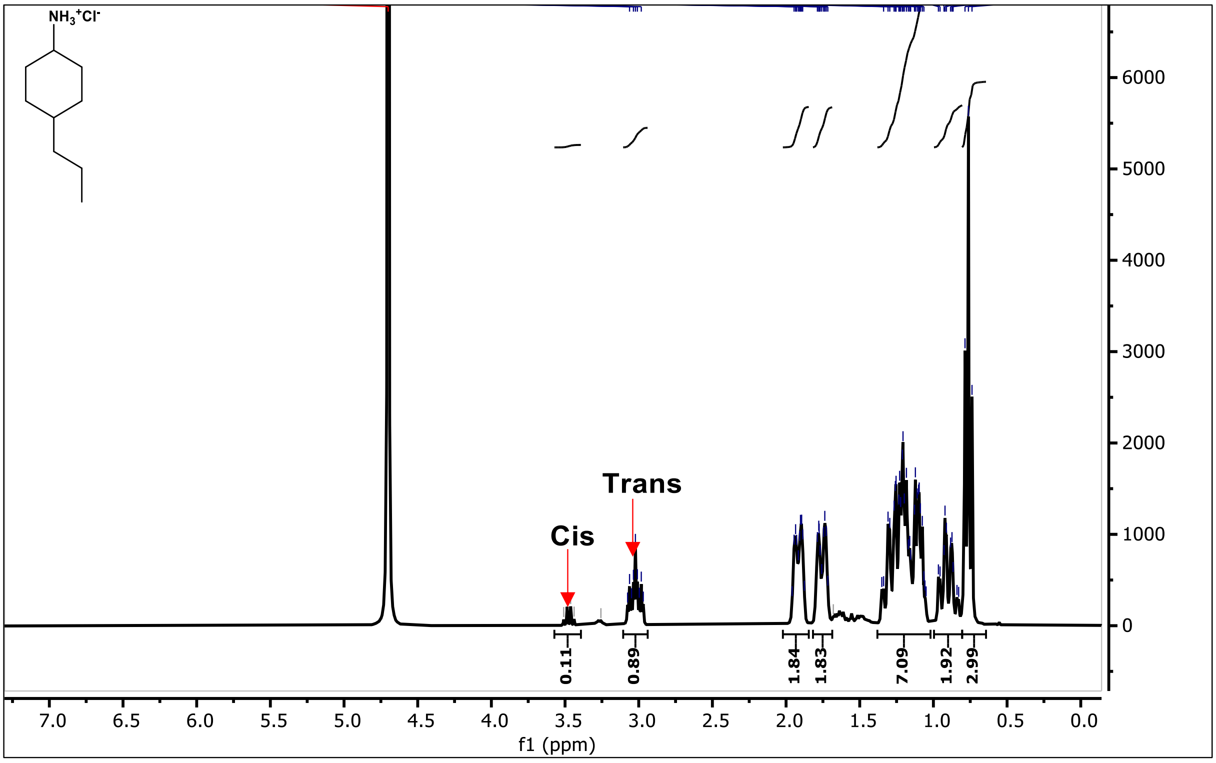

Figure S6. ^{1}H NMR spectrum of 4-propylcyclohexylamine (as its HCl salt).

5. Assigned GC-FID traces of Fraction 1 and Crude 1

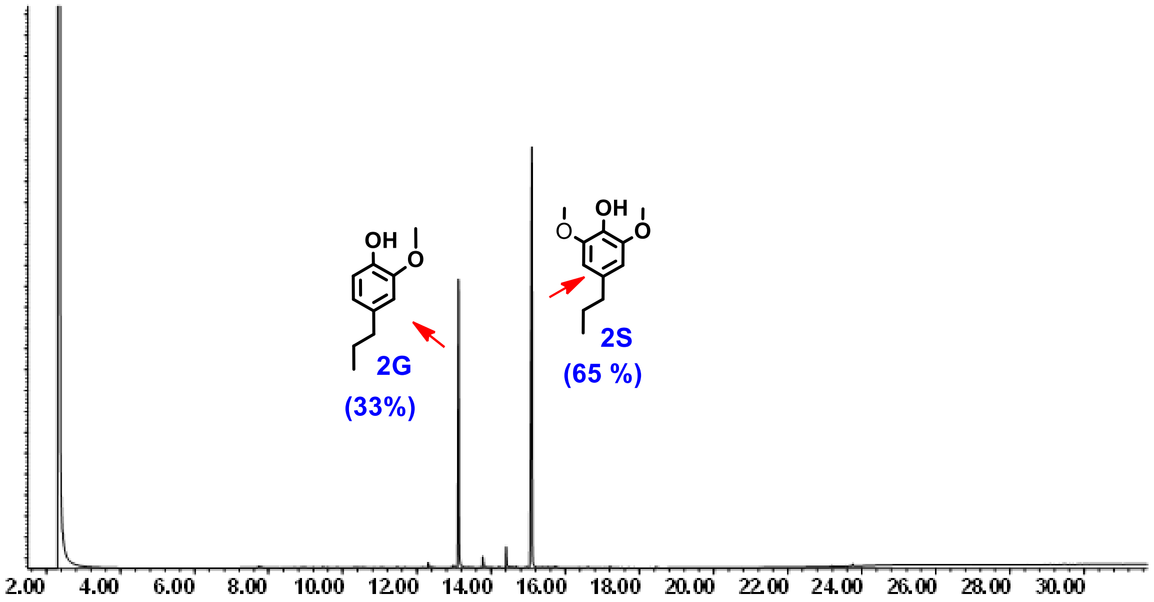

Figure S7. GC-FID trace of **Fraction 1** obtained by distillation of DCM extracted crude lignin oil, produced by RCF of birch lignocellulose**.**

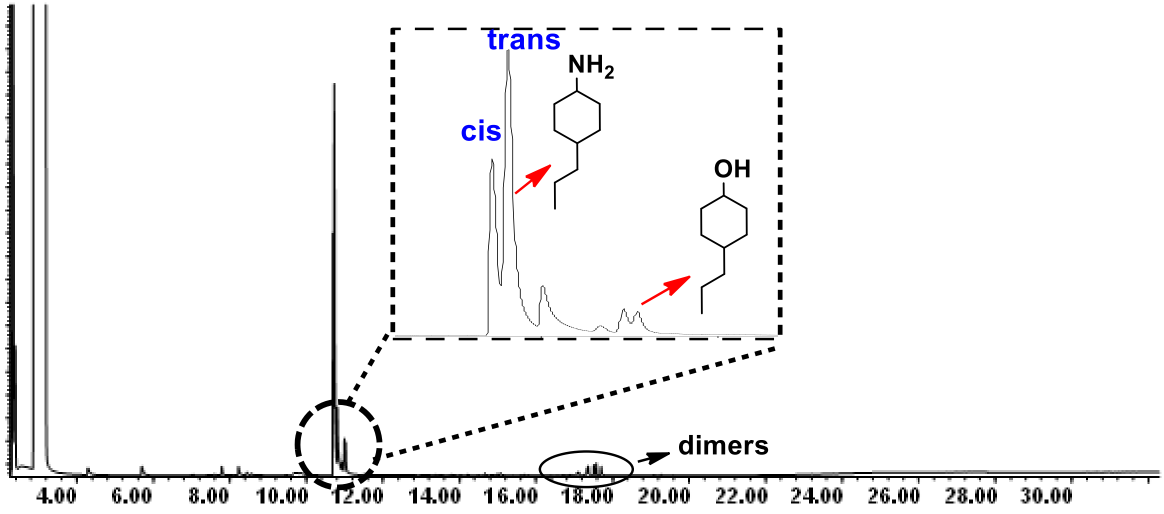

Figure S8. GC-FID trace of **Crude 1** obtained by catalytic reductive amination of **Fraction 1.**

Supplementary Note 1: CHEM21 evaluation of the greenness of the here developed process vis-à-vis an industrially relevant process to cyclohexylamine

First and foremost, we would like to note that with patents being often vague and listing broad reaction conditions, a meaningful CHEM21 analysis of an industrial method to the production of cyclohexylamine is a very challenging. Also, these industrial processes are often highly integrated with others, frequently involving heat fluxes between different processes, thus creating distinct energy economies. From our perspective the best option was a comparison of our process with the phenol-to-cyclohexylamine process, which is central to the modified Halcon (phenol ammonolysis) process (see **Scheme S1**). This may at first look odd as this process targets the formation of aniline from phenol. However, at the given high reaction temperatures, and in the presence of a catalyst and H_2, aniline is in equilibrium with cyclohexylamine. Typically, cyclohexylamine is the major product at the lower operating temperatures of this process. Additionally, we also believe that comparing a phenol *versus* a guaiacol based process is a fairer comparison to make. The below presented/discussed data (featuring **Tables S9** and **S10**) is based on numerical information extracted from Figures 6 and 7 in the literature[1] This is an older paper yet one with quite some industrial relevance/potential as it formed the exclusive basis for a recently developed techno-economic analysis of the phenol-to-aniline production process[2].

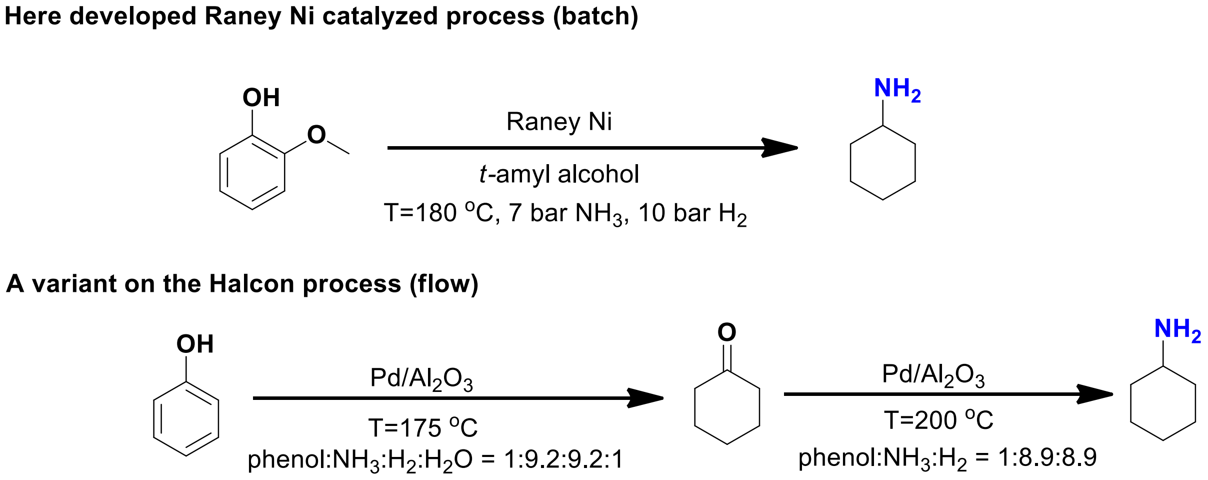

Scheme S1 An overview of the here developed process vis-à-vis an industrially relevant process to cyclohexylamine.

Comparison of the Raney Nickel catalyzed process with the modified Halcon one was performed using the CHEM21 Metrics methodology at the first path level [3], the results of which are summarized in Tables **S9** and **S10**. To the modified Halcon process the evaluation was made separately for the (consecutive) *phenol → cyclohexanone* and *cyclohexanone → cyclohexylamine* reactions – the overall credits being however easily assertible from the less performing second step. From **Tables S9** and **S10** it can be inferred that the Raney Nickel process is significantly superior to the modified Halcon process in terms of product selectivity and yield. Particularly the second step of the modified Halcon process is underperforming with clear red flags on selectivity and yield. This problem mainly relates to the formation of dicyclohexylamine and aniline as side products and not to the conversion level. The atom economy **(AE)** of the Raney Nickel process is lower than seen with the phenol-to-cyclohexylamine process, a matter which relates to the necessary loss of the methoxy group in guaiacol. It can however be argued that the side formation of methanol is actually a

beneficial process – certainly from an industrial point of view. Likewise, the loss in reaction mass efficiency **(RME)** (75.1%) relates mainly to the loss of methoxy group of guaiacol. Nonetheless, the **RME** of the Raney Nickel process is still superior to the one of the modified Halcon process and then especially when considering the poor **RME** of the second step in the modified Halcon process. The process mass intensity **(PMI)** of the Raney Nickel process is significantly higher than the ones calculated for the modified Halcon process, a matter which relates entirely to the high amount of *t*-Amyl alcohol used in the Raney Nickel process. It is however noteworthy that the MSDS sheet of *t*-Amyl alcohol does not list any of the negative Hazard codes mentioned in the CHEM21 analysis. Also, the higher amount of catalyst used does play a role, negatively influencing the **PMI** *of the reagents*. Furthermore, it is noteworthy that the modified Halcon process is operated in the gas phase without the involvement of solvents. Both processes use catalysts (green flag) but the modified Halcon processes has the advantage that it is performed in flow (green flag), whereas the Raney Nickel process features batch operation (yellow flag). From a health and safety perspective the modified Halcon process performs though much poorer than the Raney Nickel one as in addition to the use of H_2 (highly explosive) and NH_3 (environmental implications), the phenol substrate, the aniline and dicyclohexylamine by-products are toxicologically and environmentally unsustainable.

Table S9. Health & Safety assessment of the Raney Nickel method and the modified Halcon process toward cyclohexylamine.

Method	Substrate	Highly explosive	Toxic	Long term toxicity	Environmental implications
Both processes	H_2	H220			
	NH_3				H400
Modified Halcon process only	Phenol		H301, H331, H311	H341, H373	H411
	Anilline		H311, H331	H341, H351	
	Dicyclohexylamine		H301, H311		H400, H410

Table S10. The comparison of the CHEM21 metrics between the Raney Nickel method and the modified Halcon process toward cyclohexylamine.

Metric	Raney Nickel method	Flags	Variant on the Halcon method (Pd/Al_2O_3)			
			Step 1	Flags	Step 2	Flags
Yield	94%		78.2		46.8	
Conversion	94%		92		90	
Selectivity	100%		85		52	
**RME**	75.1%		81.6%		47.3%	
**AE**	79.9%		104.3%		101%	
Solvents	*t*-Amyl alcohol	N/A	Solvent-free		Solvent-free	
**PMI**	59.4		3.7		5.8	
PMI (chemicals)	6.9		3.7		5.8	
PMI (solvents)	52.5		0		0	
Catalyst	Yes		Yes		Yes	
Reactor	Batch		Flow		Flow	
Elements	Ni, Al		Pd, Al		Pd, Al	
Energy	180 °C		175 °C		200 °C	

Supplementary Note 2: Synthesis of biomass-derived *t*-amyl alcohol

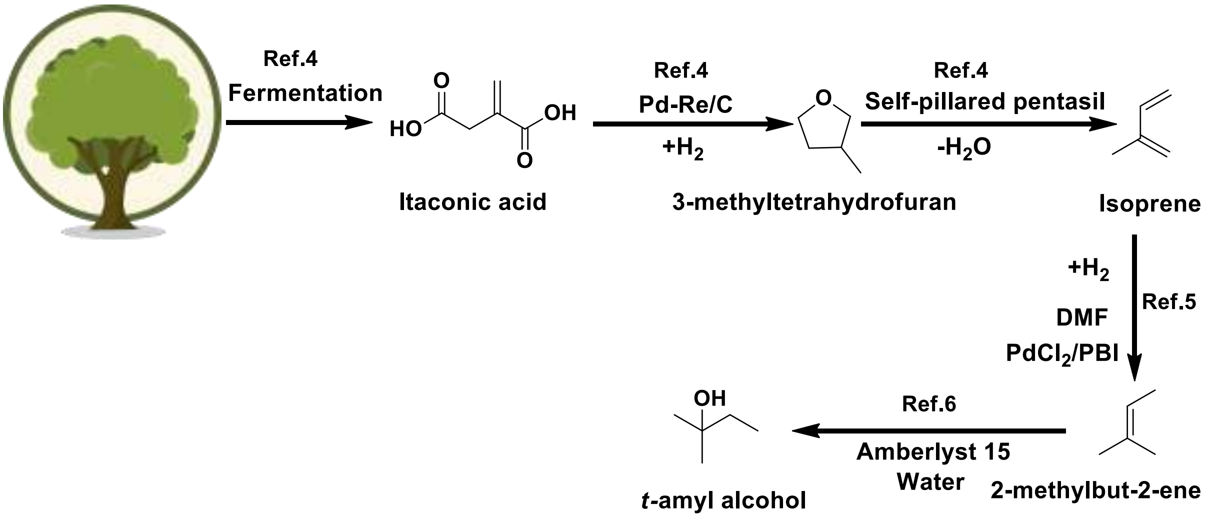

Scheme S2 A proposed reaction pathway for the production of biomass-derived *t*-amyl alcohol.

Reference

1. Y. Ono, H. Ishida, *J. Catal.* **1981**, *72*, 121-128.
2. S. Bugosen, I. D. Mantilla, F. Tarazona-Vasquez, *Heliyon* **2020**, *6*, e05778
3. C. R. McElroy, A. Constantinou, L. C. Jones, L. Summerton, J. H. Clark, *Green Chem.* **2015**, *17*, 3111-3121.
4. O. A. Abdelrahman, D. S. Park, K. P. Vinter, C. S. Spanjers, L. M. Ren, H. J. Cho, K. C. Zhang, W. Fan, M. Tsapatsis, P. J. Dauenhauer, *ACS Catal.* **2017**, *7*, 1428-1431.
5. A. Belyi, L. Chigladze, A. Rusanov, G. Gurgenidze, *Bull. Acad. Sci. USSR,* **1983**, *32*, 2400-2404.
6. B. S. Akpa, M. D. Mantle, A. J. Sederman, L. F. Gladden, *Chem. Commun.* **2005**, *21*, 2741-2743.
